# Vaccine Formulation for Infectious Diseases and Adjuvant Mechanisms of Action

**DOI:** 10.3390/vaccines9060667

**Published:** 2021-06-18

**Authors:** Heather L. Wilson, Azita Haddadi, George K. Mutwiri

**Affiliations:** 1 Vaccine and Infectious Disease Organization (VIDO), University of Saskatchewan, Saskatoon, SK S7N 5E3, Canada; george.mutwiri@usask.ca; 2 School of Public Health, University of Saskatchewan, Saskatoon, SK S7N 5E3, Canada; 3 College of Pharmacy and Nutrition, University of Saskatchewan, Saskatoon, SK S7N 5E3, Canada; azita.haddadi@usask.ca

The ultimate goal for vaccination is the generation of a safe and effective immune response that protects against diseases. Adjuvants are included to enhance and direct vaccine responses. Precisely how adjuvants and indeed vaccine formulations influence the innate immune response and the subsequent cell- and antibody-mediated arms of the adaptive immune system are being characterized and clarified. Identification of safe but potent vaccine adjuvants, particularly those directed at mucosal sites, which generate long-lasting immunological memory and are effective against intracellular and extracellular pathogens is a high priority for public health and the health of veterinary species. After they are identified, tremendous amounts of work must be done to fine-tune adjuvant production, stabilization, and purification before commercialization. We launched this special issue to highlight recent basic research in vaccine formulation for infectious diseases and assess the adjuvant mechanism of action.

The following is a synopsis of the results reported in seven original articles. Systemic or mucosal adjuvants that promote T helper 17 (Th17)-type immunity are being investigated. Chaffey et al., (2021) assessed whether an intramuscular injection of adjuvants with ovalbumin in mice could induce an antigen-specific Th17-type immune response [1]. The results showed that polyphosphazene (PCEP) alone or curdlan plus lectin did promote a Th17 response to antigens. However, local intramuscular cytokine production in the acute period and/or ex vivo stimulation of splenocytes from naïve mice exposed to these adjuvants did not produce a cytokine environment that could predict this vaccine response. Hadaddi et al. investigated the effect of a PLGA nanoparticle with a muramyl dipeptide analogue (ARC4) and a monophosphoryl lipid A analogue (ARC7) plus ovalbumin (OVA) and its effects when injected subcutaneously in mice [2]. OVA + ARC4/ARC7 nanoparticles triggered a robust and balanced Th1/Th2-type humoral response with significant anti-OVA IgA in serum and significant interferon (IFN)-γ and interleukin (IL) 17 production in splenocytes after 35 days relative to the controls. These results indicated that PLGA NPs of ARC4 + ARC7 may be inducers of a mixed Th1/Th17-type cell-mediated immune response in mice and other species. LPS is not used in vaccines due to its toxic nature, but LPS derivatives such as lipid A from commensal bacteria have the potential to be less toxic. Wang et al. examined the adjuvant activity and safety of chemically synthesized lipid A from commensal *Alcaligenes* spp. that inhabit Peyer’s patches in the gut [3]. Ex vivo analysis indicated that bone marrow-derived dendritic cells (DCs) were activated in response to lipid A and exhibited increased production of Th17-inducing cytokines IL-6 and IL-23. The mice injected subcutaneously with OVA and lipid A exhibited antigen-specific Th17 responses in the spleen with little inflammation suggesting that *Alcaligenes* lipid A may be a safe and effective Th17-type adjuvant.

Mucosal vaccines need potent mucosal adjuvants to be effective. Razim et al. developed and characterized nanoadjuvant candidates (NACs) composed of silicon oil and cationic detergents and organic solvents for intranasal application [4]. They identified NACs that are stable, do not require cold storage, and have adherence to mucin. They determined that these NACs increase the degree of OVA uptake by epithelial cells, impact the innate immune response, and induce high anti-OVA serum titers suggesting that NACs may be attractive as potential mucosal adjuvants [4].

Assessing whether FDA-approved drugs can be repurposed as vaccine adjuvants is an important way to potentially fast-track adjuvant approval. Miltefosine (MTF) is an FDA-approved anti-leishmaniasis drug. Lu et al. assessed its adjuvant potential by immunizing mice via an intraperitoneal injection with hemagglutinin from influenza followed by intranasal challenge [5]. The mice vaccinated with the MTF adjuvant exhibited increased antigen- and cell-mediated immunity as well as less pathophysiological and clinical signs of disease. Further studies will, undoubtedly, involve a more favourable route of administration. Likewise, Cao and Wang et al. assessed the immune response to vaccines formulated with varicella zoster virus glycoprotein E with immunostimulatory nucleic acids in lipid nanoparticles (LNPs), all the components of which had been approved by the FDA in other vaccines. The mice immunized intramuscularly with LNPs responded with superior antibody- and cell-mediated immunity and antigen-specific virus-neutralizing antibodies relative to the mice immunized with alum [6]. The results suggested that LNPs may be a safe and economical potential varicella and zoster vaccine candidates.

Avian metapneumovirus (aMPV) is a virus that is the causative agent of turkey rhinotracheitis in turkey flocks and swollen head syndrome in chickens leading to significant morbidity and mortality throughout China. Bao et al. performed intramuscular injections of inactivated aMPV/B strain LN16 formulated with commercially available immune-stimulating complexes (ISCOMs; Seppic) and measured the resultant immune response [7]. They determined that the vaccinated birds responded with high virus-specific serum antibodies and long-term virus-neutralizing (VN) antibodies and significant protection against aMPV/B infection with reduced virus shedding and turbinate inflammation. They suggested that this vaccine could serve as the foundation for a protective vaccine in China.

Four review articles in this issue highlight the production, purification, and formulation of interferon-based biopharmaceuticals (by Castro et al.) [8], the efficacy of natural and synthetic saponins such as QS-21 as adjuvants (by P. Wang) [9], and the efficacy of cyclic dinucleotide (CDNs) second messengers with immunomodulatory functions such as cyclic di-adenosine monophosphate (cyclic di-AMP), cyclic di-guanosine monophosphate (cyclic di-GMP), and cyclic GMP–AMP (cGAMP) as adjuvants (by Gogoi et al.) [10]. Finally, Fourie and Wilson highlighted how stress response proteins may have immunomodulation attributes making them potential adjuvants or immunotherapeutics [11].

Taken together, this special issue contains 11 relevant research and review articles focused on various aspects of the adjuvant mechanism of action and novel vaccine formulations that should be explored further for use in human and veterinary vaccines.

## Data Availability

Not applicable.
